# A “snap-shot” visual estimation of health and objectively measured frailty: capturing general health in aging older women

**DOI:** 10.1007/s40520-022-02106-y

**Published:** 2022-03-25

**Authors:** Patrik Bartosch, Linnea Malmgren, Paul Gerdhem, Jimmie Kristensson, Fiona Elizabeth McGuigan, Kristina Eva Akesson

**Affiliations:** 1 Department of Clinical Sciences Malmö, Clinical and Molecular Osteoporosis Research Unit, Lund University, 214 28 Malmö, Sweden; 2 Department of Orthopaedics, Skåne University Hospital, 205 02 Malmö, Sweden; 3 Department of Geriatrics, Skåne University Hospital, Malmö, Sweden; 4 Department of Clinical Science, Intervention and Technology, Division of Orthopedics and Biotechnology, Karolinska Institute, Solna, Sweden; 5 Department of Reconstructive Orthopedics, Karolinska University Hospital, 141 86 Stockholm, Sweden; 6 Proactive Integrated Care Research Unit, Department of Health Sciences, Faculty of Medicine, Lund University, 22100 Lund, Sweden; 7 The Institute for Palliative Care, Lund University and Region Skåne, Lund, Sweden

**Keywords:** Frailty, Visual perception, General health, Women, Community-dwelling

## Abstract

**Background:**

In clinic, a subjective visual estimation of a patient’s general health often guides interventions, yet little is known of how this assessment relates to objectively measured frailty.

**Aims:**

To characterize the relationship between these two assessments and explore the implication of discordance.

**Methods:**

The study was performed in the OPRA cohort of 75-year old community-dwelling women (*n* = 1044). Visual perception of health (VPH) was estimated within 15 s from first sight and stratified into tertiles (poor/intermediate/good health). Frailty was measured using a frailty index (FI) (scored 0.0–1.0) and stratified into tertiles: ‘*frail’* (≥ 0.22), ‘*pre-frail’* (0.13–0-21) and ‘*non-frail’* (≤ 0.12). Association between VPH and FI and with 10-year mortality was evaluated using Kaplan Meier curves and Cox proportional hazard models.

**Results:**

VPH and FI correlated, but was strongest in those perceived to be in poor health (*r*_s_ = 0.424, *p* < 0.001). Approximately half of these women were also objectively frail (53.7%). Similarly, 50.7% perceived to be in good health were also objectively non-frail. However, for one in ten, perceived health was discordant with measured frailty. Subjective and objective measures were associated with mortality, but VPH lacked discrimination in healthier looking women (*p* = 0.372) compared to FI (*p* = 0.002).

**Discussion:**

Detecting pre-frailty is important to prevent or slow the transition into a frail state. The frailest can be identified with a visual estimation, but only objective frailty assessments can reliably identity pre-frailty.

**Conclusions:**

A visual estimation of health provides valuable complementary information on health, whereas objective assessment of frailty has a broader applicability for health in aging.

**Supplementary Information:**

The online version contains supplementary material available at 10.1007/s40520-022-02106-y.

## Introduction

Frailty is a state of increased vulnerability to stressors, and intrinsically linked to age-related changes in general health. As such, it is superior to chronological age in reflecting a diminishing resilience in the aged [1]. Clinically, frailty is important because of its wide association to adverse outcomes such as hospitalisation, disability, treatment tolerance and mortality [2].

Detecting early progression of frailty or pre-frailty in the older population is important to prevent or slow down the transition into a frail state [3]. With the expected shift towards an older population, it is increasingly important to identify individuals at risk of developing frailty [4]. Early signs of frailty may be overlooked, either because of their subtle presentation, not yet visible to the eye, or at worst dismissed as normal signs of ageing [5].

To date, in the absence of a consensus on how best to measure, define and apply frailty, the clinician’s judgement is commonly used. Often necessarily brief, a visual inspection by healthcare professionals frequently serves as an estimation of an individual’s overall health [6]. This subjective “clinical eye” or visual perception of health (VPH) frequently guides further clinical decision-making albeit in conjunction with history and examination [7]. With this practice, there is nevertheless an inherent risk to misjudge a patients’ health, and therefore, refrain from administering beneficial interventions [8] or indeed, subject them to treatments or medications that would actually be harmful. Assessing frailty objectively can be time-consuming, often encompassing physical testing e.g. measuring isometric muscle strength, gait speed and balance, therefore it is easy to understand the reliance on “the clinical eye”.

It is not well established how closely subjective (i.e. VPH) and objective (i.e. frailty) estimates of general health relate to one another. A handful of studies has explored the subject in very specific patient groups, and with diverse, sometimes contradictory results [6–10]. At the population level, however, there are, to our knowledge, few or no existing studies. With limited resources in health care, it is also instrumental to know when an assessment of frailty status would actually add valuable information.

Therefore, in this exploratory study, our aim was to characterize the relationship between a subjective visual perception of health and objectively measured frailty, using a large cohort of older community-dwelling women with identical chronological age. This study explores the implications for mortality when these measures are concordant or discordant. In the study, we use a subjective visual perception of general health, which we have previously shown to be associated with fracture and 5-year mortality [11]. For comparison, we use a quantitative, cohort specific frailty index, which in the same cohort was associated with mortality, falls and fractures, [12–14].

## Materials and methods

### Subjects

This study is based on the Osteoporosis Prospective Risk Assessment (OPRA) cohort of 75 year old (75.2 ± 0.2 years) community-dwelling women. The women were randomly selected from the population register of Malmö, Sweden, at the age of 75. No exclusion criteria were applied. At baseline investigation (1995–1999), 1044 women of 1604 invited attended, giving a 65% attendance rate. At 5-year follow-up 715 attended (age 80.2 ± 0.2) and 382 (age 85 ± 0.1) at 10 years. Reasons for non-attendance are described in detail elsewhere [15]. At each visit, detailed data were collected from physical assessment (muscle strength, balance, gait, etc.), questionnaires on lifestyle and health, and blood samples [16, 17]. Date of death was acquired from the Swedish National Population Register. This study uses data from the baseline investigation only.

All procedures performed were in accordance with the ethical standards of the regional ethical review board in Lund (Dnr: 2014804), adhering to the principles of the Helsinki Declaration. All women provided written informed consent.

### Quantitative frailty assessment

Following principles suggested by Searle et al. [18], a frailty index (FI) was constructed. In brief, 13 variables associated with health, increasing with age and covering a wide spectrum of physical domains were selected. These deficits in health were used to construct the index (scored 0.0–1.0, higher score indicating higher frailty) [13]. Where an individual lacked information for a variable, the total deficits were reduced by one. The majority of variables had less than 5% missing values, while ‘self-estimated risk of falling’ and ‘diseases affecting balance’ had 13.5 and 14.9% missing, respectively (supplementary Table 1). Overall, 80% of cases had valid data for at least 12 out of the 13 variables and formal testing of the effect of missing variables on the ability of the constructed index to predict mortality showed no appreciable differences (supplementary Table 2). Furthermore, the index correlates very highly to a full 40-variable index (*r* = 0.80) [13] that had been created for the two follow-up visits [12], and both these 13- and 40-variable indices have a similar ability to predict mortality [12].

Frailty was analysed as tertiles equating to non-frail (≤ 0.12), pre-frail (0.13–0.21) and frail (≥ 0.22). We also used an empirical cut-off, where frail was defined as FI ≥ 0.25 [13].

### Subjective visual perception of health

At baseline, the women (all chronologically identically aged) had a visual perception of health status (VPH) scored within the first 15 s of sight, as detailed earlier [16]. In brief, all women were estimated by two independent healthcare professionals (aware of the participants age), using an arbitrary scale ranging from 1 to 100, where “1” represented a very healthy appearance and “100” a very unhealthy appearance. The mean value of the two scores was used in calculations. The correlation between the observers was satisfactory (*r* = 0.51–0.59, *p* < 0.0001) [16]. VPH was analysed as tertiles equating to “good”, “intermediate” and “poor” health.

The analyses in this study are based on a dataset of 1004 women for whom both FI and VPH were available. Forty women had missing VPH values; these 40 women had a higher FI compared to the cohort mean (0.31 vs 0.19, *p* < 0.001).

### Statistics

Descriptive data are presented as mean with standard deviation (SD) or median with interquartile range (IQR). Categorical variables are reported as number (*n*) and percentage (%). Association between tertiles of VPH and individual variables in the frailty index were tested using Kruskal–Wallis test, Chi-squared and ANOVA, as appropriate. Correlation between subjective and objective assessments in corresponding tertiles was tested using Spearman’s Rho.

Linear regression was used to investigate the association between VPH and FI and to what degree the VPH mirrored variation in the frailty index. To adhere to the assumptions of normality in linear regression analysis, logarithmic and square root transformations were performed for VPH and FI, respectively. The effect of significant outliers (> 3 SD, *n* = 5) was tested with or without these included.

Concordance between subjective (VPH) and objective (FI) assessments was analysed using cross tabulation and chi^2^, comparing the tertiles. Concordance was defined as being in the reciprocal tertile of both VPH and FI, i.e. visually perceived to be in good health *and* measured non-frail by frailty index or vice versa. Discordance was defined as being in the opposite tertiles of VPH and frailty, i.e. visually perceived to be in good health *but* measured as frail by frailty index or vice versa. For a visual representation of the density distribution of frailty within VPH tertiles, a spline function was used for smoothing the curves.

Using tertiles of VPH and FI, differences in 10-year mortality were assessed using Kaplan–Meier analysis with log rank. When assumptions were met, Cox regression analyses were used to calculate hazard ratios (HR). We also explored the implications for mortality when VPH and FI are not in accordance, aiming to identify in which situations VPH suffices and when an assessment of frailty adds to prediction. The results are reported without adjustments for multiple testing. For all calculations, alpha < 0.05 was considered nominally statistically significant. All calculations were performed using SPSS, IBM Corp, released 2020. IBM SPSS Statistics for Windows, Version 27.0. Armonk, NY.

## Results

### Participant characteristics stratified by visual perception of health

The characteristics of the OPRA cohort participants, stratified by subjective assessment (VPH) are presented in Table 1. Women in the poor VPH tertile had not only the highest frailty index (median 0.22; mean 0.25) but also the widest range in values (FI 0.02–0.66). Almost 40% of women who were perceived to be in poor health were objectively frail, with a FI > 0.25. By comparison, only 9% of women perceived to be in good health were objectively frail. Women in the poor VPH tertile, had higher BMI, poorer visual acuity, and more had reported having previous falls and fractures.

**Table 1:** General characteristics of the OPRA participants overall and stratified by visual perception of health tertiles

	Visual perception of health (VPH) tertile			
	Overall (*n* = 1004)	Good (*n* = 365)	Intermed (*n* = 311)	Poor (*n* = 328)
VPH range (0–100)	29.4—98.9	29.4—47.4	47.4—50.1	50.4—98.9
Frailty index (median, IQR)	0.16 (0.13)	0.12 (0.10)	0.15 (0.12)	0.22 (0.16)
Frailty index (mean, SD)	0.19 (0.11)	0.14 (0.07)	0.18 (0.09)	0.25 (0.13)
Frailty index (range 0.00–1.00)	(0.01–0.66)	(0.01–0.40)	(0.01–0.53)	(0.02–0.66)
Proportion frail (FI ≥ 0.25) % (*n*)	223 (22.2%)	33 (9.0%)	60 (19.3%)	130 (39.6%)

	Mean (SD)	Mean (SD)	Mean (SD)	Mean (SD)
BMI (kg/m^2^)	26.3 (4.19)	24.9 (3.13)	26.7 (4.06)	27.5 (4.85)
Height (cm)	160 (5.8)	161( 5.4)	160 (5.8)	160 (6.1)
Body weight (kg)	67.7 (11.5)	64.6 (8.9)	68.5 (11.0)	70.9 (13.5)
Visual acuity (average both eyes)	0.50 (0.22)	0.54 (0.22)	0.51 (0.21)	0.46 (0.23)

	*n* (%)	*n* (%)	*n* (%)	*n* (%)
Smoker (current/previous)	334 (33.6)	122 (33.5)	95 (31.0)	117 (36.2)
Alcohol (each week)	174 (17.5)	91 (25.0)	48 (15.6)	35 (10.9)
Education (elementary school level)	546 (54.5)	170 (46.4)	182 (58.9)	194 (59.1)
Fallen in previous 12 months	250 (28.2)	76 (23.1)	74 (25.1)	100 (38.3)
Any fracture between ages 50 and 75	367 (37.0)	124 (34.3)	101 (33.0)	142 (44.0)
Surgery within last 5 years	218 (23.6)	64 (19.0)	64 (21.8)	90 (30.9)

### Correlation between visual perception of health and frailty index

There was a moderate but significant correlation between visual perception of health and frailty index (*r* = 0.452; *p* < 0.001). With removal of the five outliers, the correlation increased (*r* = 0.474). Not surprisingly, the correlation between subjective and objective assessments was highest in those perceived to be in poor health (Spearman’s rho 0.403) and lowest in those perceived to be in good health (Spearman’s rho 0.147). Approximately 20% (*r*^2^ = 0.204) of the variation in VPH was explained by the frailty index.

### Concordance and discordance between visual perception of health and frailty index

**Fig. 1:**
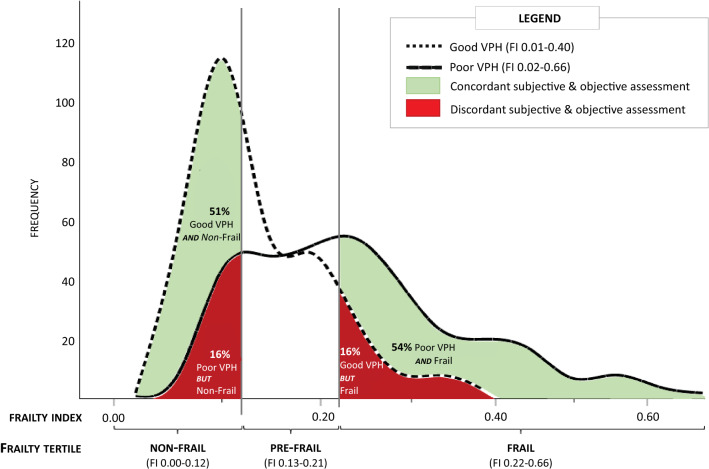
Distribution of frailty scores within the poor and good VPH tertiles. Concordance between subjective and measured assessments is highlighted in green; discordance is highlighted in red

The distribution of frailty scores within the good VPH tertile (FI 0.01–0.40) and poor VPH tertile (FI 0.02–0.66) are shown in Fig. 1, with the areas concordant and discordant for VPH-FI highlighted. No one with an FI score above 0.40 was scored in the good VPH tertile. Across all tertiles, the overall concordance was 22.3% (i.e. an individual placed in the reciprocal tertile for both VPH and FI). As can be seen in Fig. 1, approximately half of the women perceived to be in poor health were also objectively frail (53.7%). Similarly, 50.7% perceived to be in good health were also objectively non-frail. However for one in ten women, within each tertile, visually perceived health and measured frailty were discordant; specifically ~ 16% of women in the poor VPH tertile were actually non-frail and ~ 16% of women in the good VPH tertile were in fact frail.

### Visual perception of health and components of the frailty index

To understand what contributes to the snap-shot estimation of health, we analysed association between VPH and the individual components constituting the frailty index. Of these, the majority (11/13) differed by increments or decrements, stepwise across the VPH tertiles (Table 2). Those perceived to be in good health had relatively better musculoskeletal performance (gait, strength and balance) and vice versa.

**Table 2:** Visual perception of health in relation to components of the Frailty Index

Components of frailty index^a^	Visual perception of health			
	Good (*n* = 365)	Intermed (*n* = 311)	Poor (*n* = 328)	Overall
	Mean (SD)	Mean (SD)	Mean (SD)	*p* value
Gait-walking speed (m/s, 2 × 15 m) *n* = 972	1.50 (0.22)	1.30 (0.22)	1.10 (0.31)	< 0.001^d^
Gait-walking steps taken (2 × 15 m) *n* = 972	45.0 (4.4)	48.5 (5.8)	55.5 (13.7)	< 0.001^d^
Muscle strength (knee extension, Nms) *n* = 933	291 (70)	267 (76)	240 (85)	< 0.001^d^
Average time spent outdoors (h), *n* = 965	3.0 (1.2)	2.7 (1.3)	2.5 (1.3)	< 0.001^d^

	Median (IQR)	Median (IQR)	Median (IQR)	
Balance^e^ (s) *n* = 978	23.2 (15.5)	14.8 (16.5)	7.0 (16.0)	< 0.001^b^
P-CRP (mg/L), *n* = 967	1.6 (2.5)	1.9 (2.9)	2.2 (3.9)	0.001^b^
P-Creatinine (µmol/L), *n* = 972	66.2 (14.6)	66.7 (13.7)	67.1 (20.0)	0.592^b^

	*n* (%)	*n* (%)	*n* (%)	
Uses walking aid, *n* = 998	2 (0.5)	7 (2.3)	85 (26.1)	< 0.001^c^
Polypharmacy (≥ 5 medications), *n* = 1004	45 (12.3)	63 (20.3)	86 (26.2)	< 0.001^c^
High self-estimated risk of falling^f^, *n* = 876	18 (5.5)	18 (6.3)	51 (19.6)	< 0.001^c^
Have diabetes, *n* = 988	8 (2.2)	26 (8.5)	30 (9.3)	< 0.001^c^
Have had cancer/severe disease, *n* = 982	50 (13.9)	54 (17.6)	53 (16.8)	0.393^c^
Have disease affecting balance, *n* = 859	52 (16.0)	47 (16.2)	83 (33.9)	< 0.001^c^

^a^Full details of the frailty index [12, 16]

^b^Kruskal–Wallis test

^c^Chi-squared overall

^d^ANOVA

^e^Mean of time (s) for left and right leg, eyes open

^f^Values 4 or 5, in a scale 1–5

### Mortality outcomes for subjective or objectively measured health

**Fig. 2:**
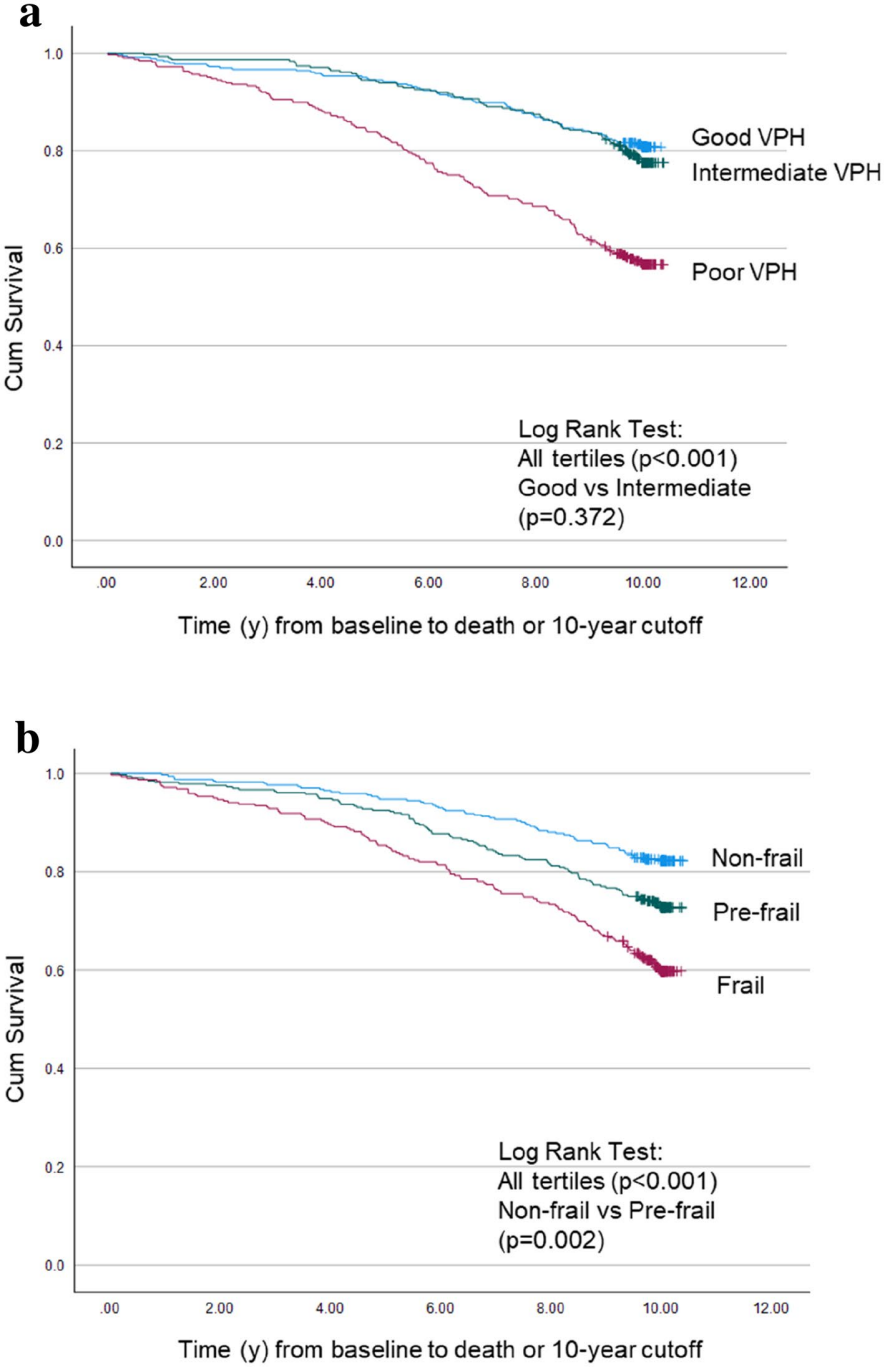
Ten-year mortality stratified by tertiles of **a** visual perception of health and **b** frailty index

Poor health, regardless of whether subjective or objective was associated with increased mortality. Being classified in the poor VPH tertile or the frail tertile was associated with higher 10-year mortality (*p* < 0.001 for both) (Fig. 2a, b); and a similar proportion were dead (43 and 40%, respectively). However, only objectively measured frailty facilitated discrimination, in terms of mortality, between the frailest in addition to the non-frail and pre-frail individuals (*p* = 0.002), while with the visual estimate mortality only differed between good and poor VPH tertiles, but not the intermediate group.

**Fig. 3:**
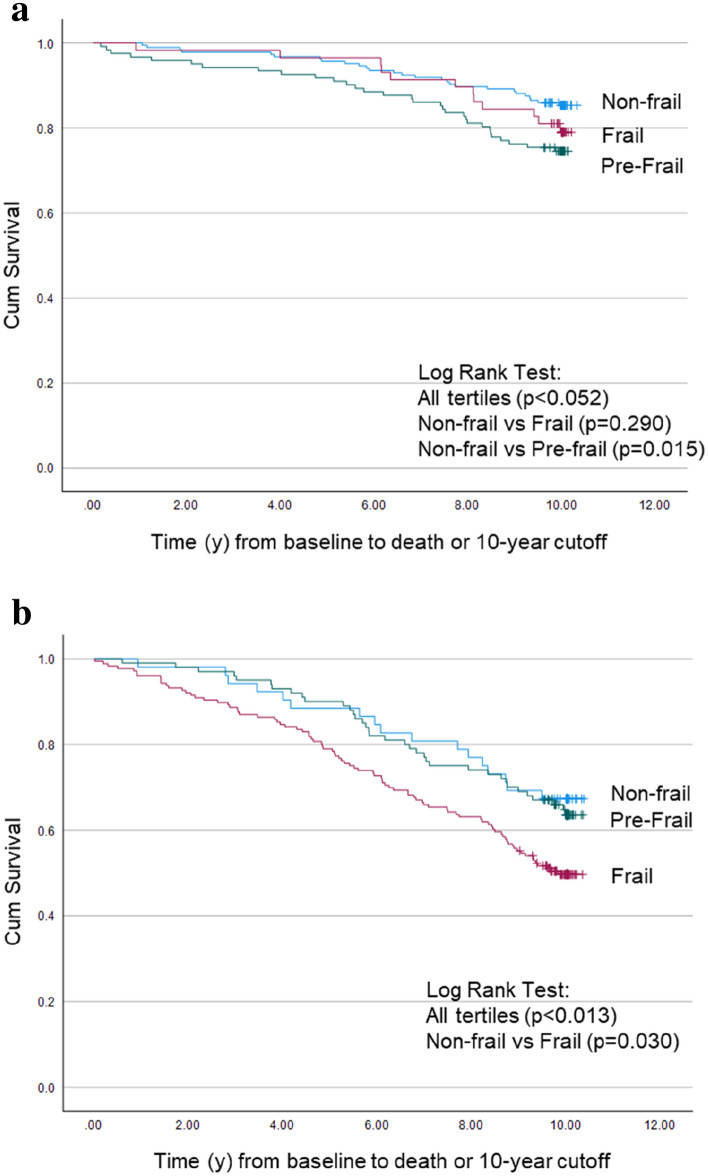
Frailty associated differences in mortality based on visual perception **a** appeared in good health, **b** appeared in poor health

Exploring the possible long-term implications when perceived and objectively measured health are not in accordance, we found that for women perceived to be in good health, mortality was similar regardless of frailty status (*p* = 0.052 overall) but was highest in the pre-frail women (*p* = 0.015) (Fig. 3a). Conversely, for those perceived to be in poor health, mortality differed by frailty status (*p* = 0.013 overall) and those who were actually non-frail by objective assessment had lower mortality (32.7 vs 50.0%; *p* = 0.023) (Fig. 3b) and lower mortality risk [HR 0.57 (0.34–0.95), *p* = 0.030] compared to those who were frail.

## Discussion

In this longitudinal study, we investigated the association between a subjective visual perception of health and an objective frailty index. The visual perception was associated with almost all individual components making up the frailty index, and subjective and objective assessments correlated. However, for one in six women, perceived and measured health was diametrically opposite, with the visual estimation being less able to identify women in the early stages of frailty. Nevertheless, both assessments were predictive of 10-year mortality.

Based on years of experience and thin-slicing, the clinician makes an instant visual assessment of a patient’s health and wellbeing. A subjective health perception can predict mortality [6, 7, 19] and in the OPRA cohort, 5-year [11] and as long as 10-year mortality was predicted by “just one look”. While there is a moderate correlation between subjective and objective assessments, the visual perception more accurately mirrors actual frailty status in those looking most obviously in poor health.

For this visual assessment, made within 15 s of first sight, more than half of those 75-year-old women classified as visually good or poor were also quantitatively non-frail or frail. Although visual perception may have its place in broadly categorizing individuals as robust or frail, it cannot reliably identify pre-frailty as many aspects may not yet be visible. Detecting the early stages of frailty is important, particularly in those that are still healthy and living independently, since pre-frailty is a major contributor to the trajectory into frailty [3, 20]. To identify and intervene with these risk individuals, as a way of delaying health declines and maintaining autonomy is now recognized as a public health and medical priority [21]. While frailty assessment is becoming increasingly common in clinic, it is not yet a standard part of the general practitioners routine, despite its potential implications for wisely choosing interventions.

We showed that almost all variables included in our frailty index, particularly walking ability, muscle strength and balance, are associated with the visual perception of health. Falls and fracture, which often affect walking ability and balance, were also more common in those that looked most obviously in poor health. This association between visual perception and mobility is also reported in other studies, with the strongest visual cue being the use of mobility aids [7, 9, 10]. Musculoskeletal competence may be the most obvious sign of frailty, but a multitude of other cues influence the judgement, such as general presentation, facial expression and coherence. In this study, around one-fifth of what an observer “sees” was explained by the frailty index, therefore, clinical observation provides valuable complimentary information. Hence, a combination of the observed status with a selection of a few of the most discriminating objective variables for frailty assessment might be the most sensible and least laborious use of the consultation time between patient and doctor.

However, it is important to recognize that the visual perception has its limitations and discordance with actual measured frailty has implications; in our study, women appearing to be in good health but who were quantifiably pre-frail had a higher mortality than might otherwise be expected. While we cannot fully explain this observation and we lack the data to address it, it indicates that only an objective assessment of frailty, using any of the available tools such as frailty index or frailty phenotype, has the sensitivity to discriminate those at pivotal junctures which would determine the individual frailty trajectory. It also argues for the need to identify frailty and intervene to maintain health, not just long-term but perhaps more relevantly in the short-term, since we have shown that in this cohort frailty is associated with falls [13] and fractures [14] within 1–3 years, all of which lead to increased frailty and disability. Given that every person’s trajectory into becoming frail is individual, treatment could entail anything from sight-tests and home assessment, to appropriate pharmaceutical interventions.

This study has several strengths, among the most important is that, the women, all are of identical chronological age. In this respect, the visual estimation is relative to the typical presentation of a 75-year old, minimising bias from the influence of chronological age on appearance. To our knowledge, this study is the first using community-dwelling participants rather than patients, to compare a subjective estimation of health to objectively measured frailty and to assess ‘real-life’ consequences of discordancy between them. Compared to others, our study has a relatively large number of participants, but being exploratory, was not designed to detect effect sizes. Paired with a randomized selection and no exclusion criteria in cohort recruitment, the findings are likely to be generalizable to a typical population of older women. Caution should of course be exercised; whether this is also generalizable to women of other ages, ethnicities, specific patient groups or men, needs to be determined.

Limitations are acknowledged, such as the difficulty to make direct comparison with available literature due to differences in estimating frailty both objectively and subjectively. It would have been advantageous to include social and cognitive factors in the frailty index, since these could enhance discrimination of pre-frailty, however, such data were not available in our cohort and furthermore, beyond the remit of the study. The moderate correlation between the VPH and FI indicates that there are other complementary cues with which the clinician makes inference, and there is undoubtedly value in using both to improve outcome [6, 9]. Finally, the small number of women who did not have VPH assessed, and were therefore excluded, had a frailty index higher than the cohort mean (0.31 and 0.19). This, in conjunction with study participants possibly being healthier than non-participants, may result in a slight, but possible selection bias towards a healthier population, a not uncommon phenomenon in elderly populations [22].

Data from this cohort suggest that a visual estimation of health can identify the most or least frail, but only by objective frailty assessment can pre-frailty be captured. Given the clinical implications from misjudging, both over- and underestimating health, an objective frailty assessment provides a more tailored method to discriminate. This allows for using the most appropriate management strategies to maintain healthy ageing.

## Supplementary Information

Below is the link to the electronic supplementary material.

Supplementary file1 (DOCX 14 kb)
