# Supplementary material for: A “snap-shot” visual estimation of health and objectively measured frailty: capturing general health in aging older women
Source: Aging Clin Exp Res. 2022 Mar 25;34(7):1663–71. doi: 10.1007/s40520-022-02106-y (PMC9246768; doi:10.1007/s40520-022-02106-y)
Supplement: Supplementary file 1 — Supplementary file1 (DOCX 14 kb) [file 40520_2022_2106_MOESM1_ESM.docx]

*Supplementary Table 1. Variables included in the Frailty Index and percent missing for each deficit (OPRA baseline, n=1044)*

|  | Frailty index (13 variables/deficits) | No. with available data | Missing Data  No. (%) |
| --- | --- | --- | --- |
| 1 | Daily physical activity | 1038 | 6 (0.6 %) |
| 2 | Average time spent outdoors | 994 | 50 (4.8%) |
| 3 | Gait - walking speed for 2 x 15m | 983 | 61 (5.8%) |
| 4 | Gait - steps taken walking 2 x 15m | 983 | 61 (5.8%) |
| 5 | Balance (2 legs, eyes closed) | 1018 | 26 (2.5%) |
| 6 | Muscle strength - knee extension | 944 | 100 (9.6%) |
| 7 | Diabetes | 1028 | 16 (1.5%) |
| 8 | Cancer | 1018 | 26 (2.5%) |
| 9 | Diseases affecting balance | 888 | 156 (14.9%) |
| 10 | Polypharmacy | 1044 | 0 (0%) |
| 11 | Self-estimated risk of falling | 903 | 141 (13.5%) |
| 12 | P-CRP | 1004 | 40 (3.8%) |
| 13 | P-Creatinine | 1011 | 33 (3.2%) |

*Supplementary Table 2. Effect of missing variables on ability of a frailty index to predict 10yr mortality*

| Test Index (No. of valid and missing variables) |  | **p-value** | **HR** | **95% CI** |
| --- | --- | --- | --- | --- |
| Any number of valid variables  *All cases included regardless of variables missing* | Q1 vs Q2 | 0.597 | 1.116 | (0.744-1.672) |
|  | Q1 vs Q3 | <0.001 | 1.967 | (1.368-2.827) |
|  | Q1 vs Q4 | <0.001 | 3.585 | (2.553-5.034) |
|  |  |  |  |  |
| Minimum 11 valid variables  *Cases with >2 variables missing (n=116) are excluded* | Q1 vs Q2 | 0.862 | 0.961 | (0.615-1.502) |
|  | Q1 vs Q3 | 0.002 | 1.880 | (1.249-2.830) |
|  | Q1 vs Q4 | <0.001 | 2.948 | (1.999-4.347) |
|  |  |  |  |  |
| Minimum 10 valid variables  *Cases with >3 variables missing (n=42) are excluded* | Q1 vs Q2 | 0.863 | 1.037 | (0.686-1.569) |
|  | Q1 vs Q3 | 0.001 | 1.852 | (1.276-2.687) |
|  | Q1 vs Q4 | <0.001 | 3.276 | (2.305-4.656) |

*Mortality risk was estimated using Cox regression and frailty index quartiles with Q1 (the healthiest quartile) as the reference category. Results are hazard ratios (HR) and 95% confidence intervals (95% CI)*
